# The effects of a pre-conception lifestyle intervention in women with obesity and infertility on perceived stress, mood symptoms, sleep and quality of life

**DOI:** 10.1371/journal.pone.0212914

**Published:** 2019-02-25

**Authors:** Lotte van Dammen, Vincent Wekker, Susanne R. de Rooij, Ben Willem J. Mol, Henk Groen, Annemieke Hoek, Tessa J. Roseboom

**Affiliations:** 1 Department of Obstetrics and Gynecology, University of Groningen, University Medical Center Groningen, Groningen, the Netherlands; 2 Department of Epidemiology, University of Groningen, University Medical Center Groningen, Groningen, the Netherlands; 3 Departments of Obstetrics and Gynecology, Amsterdam UMC at the University of Amsterdam, Amsterdam, The Netherlands; 4 Clinical Epidemiology, Biostatistics and Bioinformatics, Amsterdam UMC at the University of Amsterdam, Amsterdam, The Netherlands; 5 Amsterdam Reproduction and Development research institute, Amsterdam UMC, Amsterdam, The Netherlands; 6 Amsterdam Public Health research institute, Amsterdam UMC, Amsterdam, The Netherlands; 7 Department of Obstetrics and Gynecology, Monash University, Clayton, Victoria, Australia; Victoria University, AUSTRALIA

## Abstract

**Background:**

Obesity is an increasing problem worldwide and is associated with serious health risks. Obesity not only reduces physical health, but can also negatively affect levels of perceived stress, mood symptoms, sleep quality and quality of life (QoL), which may lead to further weight gain. We have previously shown that a pre-conception lifestyle intervention reduced weight and improved physical QoL in the short term. In the current study, we assessed the effects of this intervention in women with obesity and infertility on perceived stress, mood symptoms, sleep quality and QoL five years after randomization.

**Methods and findings:**

We followed women who participated in the LIFEstyle study. This is a multi-center randomized controlled trial comparing a six-month lifestyle intervention to improve diet and increase physical activity followed by infertility treatment, versus prompt infertility treatment. Participants were 577 women with infertility between 18 and 39 years of age with a body mass index (BMI) ≥ 29 kg/m^2^. For the current study we measured perceived stress, mood symptoms, sleep quality and QoL in 178 women five years after randomization. T-tests and linear regression models were used to assess differences between the intervention and control groups.

Five years after randomization, no differences were observed for perceived stress, mood symptoms, sleep quality and QoL between the intervention (n = 84) and control groups (n = 94). There was selective participation: women who did not participate in the follow-up had lower baseline mental QoL, and benefitted more from the intervention in terms of improved physical QoL during the original LIFEstyle intervention.

**Conclusions:**

We found no evidence that a pre-conception lifestyle intervention improved female well-being five years after randomization.

## Introduction

Obesity is an increasing health problem across the world, with rates as high as 40% among adults in the US [1]. In Europe the prevalence of overweight in 2014 has been estimated to be almost 40%, and obesity 16% [2]. Obesity not only reduces physical health [3], but may also have adverse consequences for mental health and quality of life (QoL). Obesity was associated with lower self-reported QoL in adults, and this lower QoL was even more evident in those with increased degrees of obesity [4]. Higher body weight in women was associated with higher levels of psychological and perceived stress [5]. Furthermore, depressive and anxiety disorders seem to be more prevalent in people with obesity, especially among women and those with severe obesity [6]. A bidirectional pathway might explain the causal links between obesity and depression, in which obesity leads to more depressive symptoms, and depressive symptoms lead to physiological and psychosocial alterations associated with weight gain [7]. Obesity was also associated with short sleep duration and low sleep quality, which in turn was associated with weight gain [8]. The higher prevalence of obstructive sleep apnea in adults with obesity is one of the possible causes of impaired sleep quality [8]. Low sleep quality was associated with lower QoL, more depressive symptoms and an increased cardiovascular disease risk [9–11]. These interrelations underline the importance of helping individuals not only with their obesity but also their associated mental health concerns.

Lifestyle interventions form the first step in the treatment of overweight and obesity. Optimal timing of an intervention can be important. In women, the pre-conception period provides a good opportunity to try to change lifestyle, since women are especially receptive to lifestyle advice in this time span [12]. In the LIFEstyle study, women with obesity and infertility were randomized between a lifestyle intervention group and a control group. During and directly after the six-month intervention period women in the lifestyle intervention group weighed less, presented less often with metabolic syndrome and reported better physical QoL scores compared to the control group [13]. The intervention did not improve mental QoL in the short-term.

Other lifestyle interventions aimed at weight loss have been shown to reduce the risks of associated mood problems and impaired sleep quality [14, 15]. A systematic review and meta-analysis showed that lifestyle interventions aiming at weight loss also reduced symptoms of depression [14]. In addition, two behavioral weight loss interventions demonstrated better sleep quality after a successful reduction in body weight [15]. However, these reductions were observed during the intervention period, or immediately after the intervention. Long-term effects of lifestyle interventions on weight reduction are disappointing [16], and research regarding the effects of lifestyle interventions on long-term well-being and sleep quality is scarce. Such research is highly relevant as mental well-being and good sleep quality are important determinants of adequate daily life functioning, and could possibly increase motivation to lose weight and lead to better weight maintenance [17, 18].

We investigated whether the pre-conception LIFEstyle intervention in women with obesity and infertility led to improved levels of perceived stress, mood symptoms, sleep quality and QoL five years after randomization.

## Methods

We followed women approximately five (range = 3–8) years after participation in a pre-conception lifestyle intervention trial [19], the LIFEstyle study [20, 21]. The LIFEstyle study was a randomized controlled trial (RCT) carried out in 23 hospitals in the Netherlands between 2009–2012 (Netherlands Trial Registry (NTR 1530)). The study was approved by the medical ethics committee of the University Medical Center Groningen (UMCG) (METc code: 2008/284) and conducted following the principles of the Declaration of Helsinki. Women were eligible for the LIFEstyle study if they had a body mass index (BMI) of ≥ 29 kg/m^2^, were between 18 and 39 years of age and had infertility. The inclusion criterion for BMI was chosen based on previous research reporting declined pregnancy chances with a BMI of ≥ 29 kg/m^2^ [22]. Infertility was defined as: chronic anovulation; or, unsuccessful attempts to conceive for at least 12 months with an ovulatory cycle. In total 577 women with obesity and infertility provided written informed consent and were randomized between a six-month lifestyle intervention group, and a control group. The six-month lifestyle intervention aimed at 5–10% weight loss or a BMI < 29 [3] through a healthy diet, increased physical activity and behavioral modification. Behavior change was targeted by means of motivational counselling, which was effective in reducing weight in previous research [23, 24]. The motivational counselling was directed toward:

- Awareness of actual lifestyle leading to obesity.
- Counselling healthy lifestyle measures: the effect of healthy lifestyle, including diet and physical activity, in relation to subfertility and pregnancy chances, pregnancy complications and perinatal outcomes.
- Formulating individualized goals regarding diet and physical activity embedded in a "patient contract". During the intervention individual goals were evaluated, feedback was given and goals were adapted if necessary. The coaches also provided information about weight regain and relapse, and how to continue after a setback.

Trained coaches guided the lifestyle intervention, which consisted of six outpatient visits and four telephone consultations during the six-month intervention period. The intervention group received infertility treatment as indicated after the six-month intervention period, or if the weight loss goal of the intervention was reached before the six-month period ended. The control group received no lifestyle or body-weight related intervention, only prompt infertility treatment. The results of the effect of the lifestyle intervention on fertility and pregnancy outcomes have been published previously [21]. Rates of vaginal birth of a healthy singleton at 37 weeks of gestation or more, which was the primary outcome of the study, were not higher in the intervention group compared to the control group [21]. The lifestyle intervention significantly reduced weight and led to improved cardiometabolic health and physical QoL in the short-term [13].

Of the 577 women who were randomized, three women withdrew informed consent and 10 were lost to follow-up during the first 24 months of the study, leaving a total of 564 women who completed the original study. For 14 of these women, contact information was unknown at the time of the follow-up study, or they emigrated, leaving 550 available for the present follow-up. Eligible women for whom contact information was known (n = 550) received an information leaflet at home regarding the follow-up. Women were contacted several times by mail and telephone in order to include them in the follow-up. The five year follow-up consisted of a questionnaire packet with items regarding QoL and symptoms of depression and anxiety. This questionnaire also included items regarding education level, self-reported weight, and parity; a detailed description of the questionnaire packet is provided below. The questionnaire was filled out online or a printed copy was mailed. All eligible women were also invited in a second information leaflet to participate in a separate visit involving physical measurements and an additional questionnaire packet in which perceived stress and sleep quality were measured with detailed description provided below. The primary focus of the physical measurements was to assess anthropometrics and cardiometabolic health. The median number of months between the basic questionnaire and additional visit/questionnaire was five months.

Baseline information at the time of randomization was available for age, ethnic background, education level, BMI, diagnosis of polycystic ovary syndrome (PCOS), whether the woman had ever conceived and QoL. During the entire original trial, levels of perceived stress, symptoms of depression and anxiety and sleep quality were not assessed.

### Perceived stress

The Dutch version of the 10-item perceived stress scale (PSS) was used to measure perceived stress [25]. This questionnaire has been labelled a valid and internally consistent measure of perceived stress [26, 27]. A total score was calculated as a sum score from the 10 items.

### Mood symptoms

For the assessment of symptoms of depression and anxiety, the Dutch version of the hospital anxiety and depression scale (HADS) was used [28]. This 14-item questionnaire has demonstrated good sensitivity and specificity [29, 30]. Seven items measure symptoms of depression, and the other seven items measure symptoms of anxiety. The total score for depression and anxiety is the sum score of these items.

### Sleep quality

The Dutch version of the Pittsburgh sleep quality index (PSQI) was used to measure sleep quality. This questionnaire consists of 19 items, with seven subscales from which a total score can be derived, with higher scores indicating worse sleep quality [31]. The subscales are ‘subjective sleep quality’ based on one question assessing sleep quality, ‘sleep duration’, based on one question assessing sleep duration, ‘sleep disturbances’ based on nine types of sleep disturbances, ‘sleep latency’ based on questions assessing the average time it takes to fall asleep, ‘day dysfunction’, based on two questions regarding daytime functioning, ‘habitual sleep efficiency’, based on the time spent in bed sleeping, and ‘use of sleeping medication’, based on one question assessing the use of sleep medication. The PSQI has shown good validity and internal consistency [31, 32].

### Quality of life

We measured QoL with the Dutch version of the short form 36 (SF-36), a validated questionnaire with 36 questions regarding physical and mental QoL [33–35]. Higher scores indicate better QoL. The subscales ‘physical functioning’, based on the performance of physical activity without limitations due to health, ‘role limitations due to physical problems’, based on problems with work or daily activities as a result of physical health, ‘bodily pain’, based on physical pain or limitations due to pain, ‘general health’, based on the evaluation of personal health and ‘vitality’, based on energy levels, form the physical component summary (PCS) of the QoL. The subscales ‘social functioning’, based on the ability to perform normal social activities, ‘role limitations due to emotional problems’, based on problems with work or daily activities resulting from emotional problems, ‘mental health’, based on feeling peaceful, happy and calm, and ‘vitality’, based on energy levels, form the mental component summary (MCS) of the QoL. Baseline SF-36 data at time of randomization were also available.

### Statistical analyses

Comparisons of follow-up characteristics for the intervention and control groups were performed. Potential selection bias was assessed by comparing the baseline characteristics between participants and non-participants in the follow-up. Both these assessments were performed with *t*-tests, Fisher-Freeman-Halton tests and chi-square tests, after the characteristics and outcome scores were checked for normality. The comparisons between the intervention and control groups for levels of perceived stress, symptoms of depression and anxiety and sleep quality were analyzed with *t*-tests. Changes in the subscales and component scores of QoL were calculated and used as the outcome in multivariable regression models comparing the intervention and control groups, covarying for the baseline measurement of that outcome. Results were considered statistically significant if a *p* value was < 0.05. Statistical analyses were performed using IBM SPSS version 24.0 (Armonk, NY, USA).

## Results

### Participant flow and characteristics

**Fig 1:**
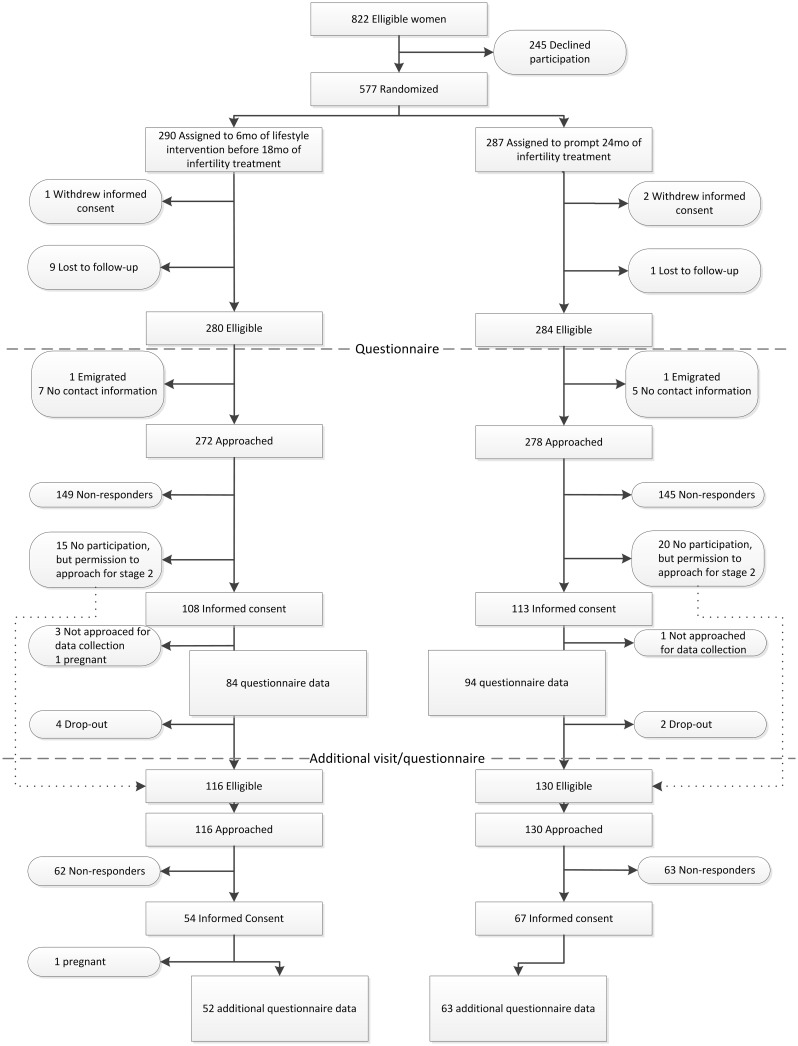
Flowchart of participants.

Fig 1 shows the flow of the 550 women invited to take part in the follow-up study. Of the 216 women who provided informed consent to fill out the first questionnaire packet, 38 women failed to do so in the end. A total of 178 (32%) women participated in the follow-up, of whom 84 women had been allocated to the intervention group and 94 women to the control group. They filled out the questionnaires regarding depression, anxiety and QoL. Of the 120 women who provided informed consent to fill out the additional questionnaire, 5 women did not actually do this. The additional questionnaire measuring perceived stress and sleep quality was filled out by 115 women (21%), of whom 52 had been in the intervention group and 63 in the control group. In Table 1 the differences at baseline between the participants and non-participants in the follow-up are shown.

**Table 1:** Comparison of baseline characteristics of participants and non-participants in the follow-up of the LIFEstyle study.

	Participants (n = 178)	Non-participants in the follow-up (n = 396)	*p* value
Age in years (mean, SD)	30.0 (4.2)	29.7 (4.7)	0.41
Western European ethnicity (n, %)	169 (94.9)	333 (84.1)	< 0.01
Education level (n, %)			0.07
- Primary school	5 (2.9)	22 (5.9)	
- Secondary education	33 (19.0)	98 (26.1)	
- Intermediate vocational education	96 (55.5)	170 (45.2)	
- Higher vocational education and university	39 (22.5)	86 (22.9)	
Body mass index in kg/m^2^ (mean, SD)	36.0 (3.2)	36.1 (3.5)	0.62
Polycystic ovary syndrome (n, %)	74 (41.6)	127 (32.2)	0.03
Never conceived at baseline (n, %)	114 (64.0)	255 (64.6)	0.91
**Baseline quality of life (QoL) measures**			
Physical functioning (mean, SD)	84.3 (15.5)	83.8 (17.4)	0.76
Role limitations due to physical problems (mean, SD)	80.9 (36.6)	83.7 (32.6)	0.40
Bodily pain (mean, SD)	82.4 (22.8)	81.8 (23.7)	0.82
General health (mean, SD)	66.3 (17.5)	63.4 (18.4)	0.10
Vitality (mean, SD)	61.0 (16.0)	58.1 (17.4)	0.08
Social functioning (mean, SD)	86.4 (15.9)	81.3 (21.6)	< 0.01
Role limitations due to emotional problems (mean, SD)	90.0 (25.4)	80.7 (34.9)	< 0.01
Mental health (mean, SD)	77.5 (12.2)	72.6 (16.0)	< 0.01
Physical Component Summary (PCS) (mean, SD)	49.3 (9.0)	50.2 (9.3)	0.36
Mental Component Summary (MCS) (mean, SD)	52.1 (7.4)	48.5 (10.5)	< 0.01

Abbreviations: SD = standard deviation.

Non-participants in the follow-up were less often of Western European descent, had PCOS less often, and had lower scores on the QoL subscales ‘social functioning’, ‘role limitations due to emotional problems’ and ‘mental health’, indicating worse mental QoL. The mental component summary was also significantly lower at baseline for non-participants in the follow-up. The subscales ‘social functioning’ and ‘mental health’ were significantly better at baseline in the intervention group of participants, and the subscale ‘role limitations due to emotional problems’ and the mental component summary were significantly better at baseline in both the intervention and control groups of participants compared to non-participants in the follow-up. Furthermore, the short-term improvement in physical QoL in the intervention group was predominantly among women who did not participate in the follow-up (*p* = 0.01 for non-participants in the follow-up, *p* = 0.16 for follow-up participants). Baseline characteristics were similar between participants who filled out the first questionnaire packet (n = 178) and participants who filled out the additional questionnaire packet (n = 115) (*p* ≥ 0.05). The participant characteristics at follow-up were similar between the intervention and control groups, as shown in Table 2. Self-reported BMI and weight loss based on self-reported weight at follow-up were similar for the intervention and control groups.

**Table 2:** Participant characteristics at follow-up.

	Lifestyle intervention group (n = 84)	Control group (n = 94)	*p* value
Age in years (mean, SD)	35.1 (4.2)	34.8 (4.5)	0.64
Western European ethnicity* (n, %)	78 (92.9)	91 (96.8)	0.23
Education level (n, %)			0.09
- No education	1 (1.2)	0 (0)	
- Primary school	0 (0)	4 (4.3)	
- Secondary education	21 (25.0)	14 (14.9)	
- Intermediate vocational education	41 (48.8)	53 (56.4)	
- Higher vocational education and university	21 (25.0)	23 (24.5)	
Self-reported body mass index in kg/m^2^ (mean, SD)	34.4 (5.1)	34.5 (5.0)	0.83
Weight loss in kg based on self-reported weight (mean, SD)	-5.2 (13.0)	-3.8 (14.3)	0.49
Polycystic ovary syndrome *, diagnosed by Rotterdam 2003 criteria (n, %)	32 (38.1)	42 (44.7)	0.37
No child at follow-up (n, %)	18 (21.4)	15 (16.0)	0.35

* Measured at baseline.

Abbreviations: SD = standard deviation.

### Perceived stress, mood symptoms, sleep quality and quality of life

Tables 3 and 4 show the results of the lifestyle intervention on levels of perceived stress, mood symptoms, and sleep quality. No differences were observed between the intervention and control group for levels of perceived stress, symptoms of depression and anxiety, or sleep quality. The effect sizes for these outcomes were all small (Cohen’s *d*: 0.005 to 0.11). In Table 5, the results for QoL also showed no difference in change from baseline to follow-up between the intervention and control groups, and effect sizes again were small (Cohen’s *d*: 0.036 and 0.176). No differences were observed for any of the outcomes when participants were divided between women with a child, and women who never conceived (S1, S2 and S3 Tables).

**Table 3:** Symptoms of depression and anxiety measured by the HADS and perceived stress measured by the PSS five years after lifestyle intervention according to allocation status.

	Intervention group	n	Control group	n	Mean difference (95% confidence interval)	*p* value
Symptoms of depression	7.7 (0.4)	84	7.7 (0.3)	94	0.02 (-1.0 to 1.0)	0.97
Symptoms of anxiety	8.0 (0.4)	84	8.2 (0.3)	94	-0.2 (-1.3 to 0.8)	0.68
Perceived stress levels	14.4 (0.9)	52	13.7 (0.6)	63	0.7 (-1.5 to 2.8)	0.54

Data are given as means (Standard Error); the mean difference between intervention and control groups was assessed with student t-tests. Scores can range from 0 to 21 for symptoms of depression and anxiety, and from 0 to 40 for perceived stress levels with higher scores indicating more symptoms.

**Table 4:** Sleep quality measured by the PSQI, five years after lifestyle intervention according to allocation status.

	Intervention group (n = 52)	Control group (n = 63)	Mean difference (95% confidence interval)	*p* value
Sleep quality total score	5.4 (0.5)	5.3 (0.4)	0.1 (-1.2 to 1.4)	0.86
- Subjective sleep quality	1.0 (0.1)	1.1 (0.1)	-0.01 (-0.3 to 0.3)	0.94
- Sleep duration	0.4 (0.1)	0.5 (0.1)	-0.06 (-0.4 to 0.2)	0.71
- Sleep disturbances	1.2 (0.1)	1.2 (0.1)	-0.01 (-0.2 to 0.2)	0.96
- Sleep latency	1.1 (0.1)	1.1 (0.1)	0.03 (-0.4 to 0.4)	0.88
- Day dysfunction	0.8 (0.1)	0.7 (0.1)	0.02 (-0.2 to 0.3)	0.88
- Habitual sleep efficiency	0.8 (0.1)	0.8 (0.1)	-0.03 (-0.4 to 0.4)	0.87
- Use of sleeping medication	0.1 (0.1)	0.2 (0.1)	-0.04 (-0.3 to 0.2)	0.71

Data are given as means (Standard Error); the mean difference between intervention and control groups was assessed with student t-tests. The total score ranges from 0 to 21 where higher scores represent worse sleep quality.

**Table 5:** Quality of life measured by the SF-36, change from baseline scores five years after lifestyle intervention according to allocation status.

	Intervention group (n = 65)	Control group (n = 84)	Mean difference (95% confidence interval)	*p* value
Physical functioning	0.7 (2.0)	-1.0 (1.8)	1.7 (-3.7 to 7.1)	0.54
Role limitations due to physical problems	-1.8 (4.1)	-8.4 (3.7)	6.6 (-4.5 to 17.6)	0.24
Bodily pain	-6.0 (3.0)	-10.1 (2.7)	4.1 (-3.9 to 12.1)	0.31
General health	-17.6 (0.4)	-17.8 (0.4)	-0.2 (-0.9 to 1.4)	0.71
Vitality	-1.8 (2.3)	-7.3 (2.1)	-5.4 (-0.7 to 11.5)	0.08
Social functioning	-3.3 (2.4)	-4.3 (2.2)	1.0 (-5.4 to 7.4)	0.76
Role limitations due to emotional problems	-6.4 (3.9)	-6.7 (3.5)	0.3 (-10.1 to 10.7)	0.95
Mental health	-3.0 (1.7)	-2.4 (1.5)	-0.6 (-5.1 to 4.0)	0.81
**Physical Component Summary (PCS)**	-2.5 (1.0)	-3.9 (0.9)	1.5 (-1.3 to 4.2)	0.29
**Mental Component Summary (MCS)**	-1.6 (1.1)	-1.9 (1.0)	0.3 (-2.6 to 3.2)	0.83

Data are given as mean change scores (Standard Error), adjusted for baseline measurement; the mean difference between intervention and control group was assessed in multivariable regression models. The scores range from 0 to 100, with higher scores indicating better QoL.

## Discussion

In this five year follow-up visit of a pre-conception lifestyle RCT in women with obesity and infertility, we were unable to detect any effects on levels of perceived stress, symptoms of depression and anxiety, sleep quality and QoL among those who participated in the follow up.

There are several potential explanations for the null findings in our study. First, a pre-conception lifestyle intervention might not lead to improved levels of perceived stress, mood symptoms, sleep quality and QoL. On the other hand, effects of the lifestyle intervention on well-being and sleep quality might only be present if weight loss is maintained over time. Furthermore, this study was not powered for studying effects on mental health five years after randomization. The effects sizes we observed were very small to small, indicating that even with sufficient power the effect sizes would be small. Relatively small differences cannot be detected with the current sample size (post-hoc power QoL: 33%). Lastly, the intervention might have been more successful in improving mental well-being, if specific factors had been incorporated in the intervention, including mindfulness or cognitive behavioral therapy [36, 37], or if group sessions had been combined with the individual coaching sessions [38].

Only 32% of the women who were invited to participate, filled out the first questionnaire packet, and an even smaller group filled out the additional questionnaire (21%). Women who participated in the follow-up more often had PCOS, which limits the generalizability of our results, and may have led to an underestimation of the effect of the intervention on mental well-being, since PCOS is linked to impaired mental well-being [39]. Women in the control group were more likely to participate in the follow-up, possibly because they did not participate in the intervention and thus did not experience the follow-up as a checkup of intervention effectiveness, and current lifestyle and body composition examination. Mental QoL at baseline was lower among women who did not participate in the follow-up, indicating that women with lower mental well-being were less interested in participation or less able to participate in follow-up measurements, possibly because they were less motivated. This baseline difference in mental QoL was more pronounced in the intervention group, which might have led to an underestimation of the long-term effect of the lifestyle intervention on mental QoL. Moreover, the short-term positive effect of the lifestyle intervention on physical QoL was observed predominantly among women who did not participate in the follow-up, and not among women who did participate in the follow-up. This indicates that a specific subgroup of women participated in the follow-up, mainly those women who did not benefit from the intervention in terms of improved physical QoL during the LIFEstyle intervention.

Our null findings are not in line with a previous study examining long-term effects of a lifestyle intervention. In the Look AHEAD trial, an intensive lifestyle intervention of individual and group meetings in individuals with overweight or obesity with type 2 diabetes led to improved mood in both short- and long- terms [38]. The lifestyle intervention also led to long-term maintained weight loss [40], which was not the case in the selective group that participated in our follow-up, and could partially explain our findings. Furthermore, the Look AHEAD intervention was of longer duration (eight years), compared to the six month LIFEstyle intervention, although other evidence suggests intervention duration is not related to the magnitude of change in symptoms of depression [14]. Besides weight and mood symptoms, QoL was also assessed in the Look AHEAD trial, and over time a reduction was observed in physical and mental QoL, with a smaller reduction in the intervention group, compared to the control group [38]. QoL also reduced over time in the participants of our study, but this decline was not significantly different between the intervention and control groups. The minimal clinically important difference for QoL measured by the SF-36 has been estimated to be 2–4 points, while in our results a difference in decline between the intervention and control groups was approximately 1.5 points for physical QoL and 0.3 point for mental QoL. Hence, even with sufficient power, the long-term differences in QoL between the intervention and control groups would not have been clinically relevant. The reduction of QoL over time has been documented in previous research, also among women in the same age group as our sample. This reduction has been associated with increasing age, suggesting a relation with an increased prevalence of disease over time [41, 42].

We compared the mean scores of all outcomes to normative general population values. Overall, levels of perceived stress, depression, anxiety, sleep quality and QoL were comparable to normative values [31, 43–46]. This suggests our questionnaire data are valid when compared to normative values.

A strength of this study is the assessment of QoL at baseline and follow-up, providing information about changes in QoL over time. Limitations include selective participation and the lack of baseline and short-term data for perceived stress, mood symptoms and sleep quality. As a result we do not know if the intervention had any short-term effects on these outcomes.

Despite promising short-term effects on body weight and QoL, we were unable to detect effects of a lifestyle intervention on levels of perceived stress, mood symptoms, sleep quality and Qol in women with obesity and infertility five years after randomization. However, future research regarding lifestyle intervention should investigate long-term effects on these outcomes, and whether effects on mental well-being depend on weight loss, since mental well-being and good sleep quality are determinants of daily functioning and may lead to improved cardiometabolic health. In future research regarding lifestyle interventions, high attrition rates should be anticipated in power calculations, consequently increasing the number of initial participants. Furthermore, since selective participation leads to difficulties in the evaluation of the effectiveness and the generalizability of the results, novel strategies are needed to engage study subjects to participate in follow-up measurements.

## Supporting information

S1 Table Mean scores (Standard Error) of symptoms of depression, anxiety and perceived stress, the mean difference between women with at least one child and women with no children, assessed with student t-tests. Scores can range from 0 to 21 for symptoms of depression and anxiety, and from 0 to 40 for perceived stress levels with higher scores indicating more symptoms.
(DOCX)

S2 Table Mean scores (Standard Error) of sleep quality, the mean difference between women with at least one child and women with no children, assessed with student t-tests. The total score ranges from 0 to 21 where higher scores represent worse sleep quality.
(DOCX)

S3 Table Mean scores (Standard Error) on questionnaires assessing quality of life, mean difference between women with at least one child and women with no children, assessed with student t-tests. The scores range from 0 to 100, with higher scores indicating better QoL.
(DOCX)

S1 File Data set. (SAV)
