# Supplementary material for: The effects of a pre-conception lifestyle intervention in women with obesity and infertility on perceived stress, mood symptoms, sleep and quality of life
Source: PLoS One. 2019 Feb 25;14(2):e0212914. doi: 10.1371/journal.pone.0212914 (PMC6388912; doi:10.1371/journal.pone.0212914)
Supplement: S1 Table — Scores can range from 0 to 21 for symptoms of depression and anxiety, and from 0 to 40 for perceived stress levels with higher scores indicating more symptoms. (DOCX) [file pone.0212914.s001.docx]

|  | **Women with at least one child** | **n** | **Women with no children** | **n** | **Mean difference (95% confidence interval)** | ***p* value** |
| --- | --- | --- | --- | --- | --- | --- |
| Symptoms of depression | 7.7 (0.3) | 144 | 7.7 (0.7) | 34 | 0.01 (-1.3 to 1.3) | 0.99 |
| Symptoms of anxiety | 8.1 (0.3) | 144 | 8.1 (0.8) | 34 | -0.01 (-1.4 to 1.3) | 0.98 |
| Perceived stress levels | 13.6 (0.6) | 83 | 15.3 (1.5) | 21 | 1.8 (-1.0 to 4.5) | 0.20 |
