# Supplementary material for: The effects of a pre-conception lifestyle intervention in women with obesity and infertility on perceived stress, mood symptoms, sleep and quality of life
Source: PLoS One. 2019 Feb 25;14(2):e0212914. doi: 10.1371/journal.pone.0212914 (PMC6388912; doi:10.1371/journal.pone.0212914)
Supplement: S2 Table — The total score ranges from 0 to 21 where higher scores represent worse sleep quality. (DOCX) [file pone.0212914.s002.docx]

|  | **Women with at least one child (n=83)** | **Women with no children (n=21)** | **Mean difference (95% confidence interval)** | ***p* value** |
| --- | --- | --- | --- | --- |
| Sleep quality total score | 5.1 (0.3) | 5.1 (0.9) | -0.04 (-1.6 to 1.5) | 0.95 |
| - Subjective sleep quality | 1.1 (0.1) | 0.9 (0.2) | -0.2 (-0.6 to 0.2) | 0.26 |
| - Sleep duration | 0.4 (0.1) | 0.4 (0.2) | 0.01 (-0.4 to 0.4) | 0.96 |
| - Sleep disturbances | 1.1 (0.1) | 1.2 (0.1) | 0.1 (-0.1 to 0.4) | 0.28 |
| - Sleep latency | 1.1 (0.1) | 0.9 (0.2) | -0.1 (-0.6 to 0.4) | 0.57 |
| - Day dysfunction | 0.7 (0.1) | 1.0 (0.2) | 0.3 (-0.04 to 0.6) | 0.09 |
| - Habitual sleep efficiency | 0.7 (0.1) | 0.7 (0.2) | -0.01 (-0.5 to 0.4) | 0.97 |
| - Use of sleeping medication | 0.1 (0.1) | 0.2 (0.2) | 0.1 (-0.2 to 0.4) | 0.48 |
