# Supplementary material for: The effects of a pre-conception lifestyle intervention in women with obesity and infertility on perceived stress, mood symptoms, sleep and quality of life
Source: PLoS One. 2019 Feb 25;14(2):e0212914. doi: 10.1371/journal.pone.0212914 (PMC6388912; doi:10.1371/journal.pone.0212914)
Supplement: S3 Table — The scores range from 0 to 100, with higher scores indicating better QoL. (DOCX) [file pone.0212914.s003.docx]

|  | **Women with at least one child (n=141)** | **Women with no children (n=34)** | **Mean difference (95% confidence interval)** | ***p* value** |
| --- | --- | --- | --- | --- |
| Physical functioning | 85.0 (1.5) | 84.4 (2.2) | -0.6 (-6.9 to 5.7) | 0.85 |
| Role limitations due to physical problems | 76.2 (3.0) | 75.7 (6.3) | -0.5 (-14.0 to 13.0) | 0.94 |
| Bodily pain | 75.9 (2.1) | 72.9 (3.5) | -3.0 (-12.1 to 6.1) | 0.51 |
| General health | 48.5 (0.3) | 48.8 (0.7) | 0.3 (-1.1 to 1.8) | 0.64 |
| Vitality | 55.9 (1.6) | 57.9 (4.4) | 2.0 (-7.4 to 11.4) | 0.67 |
| Social functioning | 83.6 (1.7) | 80.5 (3.6) | -3.1 (-10.6 to 4.5) | 0.42 |
| Role limitations due to emotional problems | 85.6 (2.6) | 76.5 (7.0) | -9.1 (-24.1 to 5.9) | 0.23 |
| Mental health | 74.6 (1.3) | 74.2 (2.3) | -0.4 (-6.1 to 5.4) | 0.91 |
| **Physical Component Summary (PCS)** | 46.3 (0.7) | 46.5 (1.2) | 0.2 (-3.0 to 3.4) | 0.91 |
| **Mental Component Summary (MCS)** | 50.2 (0.8) | 49.0 (1.8) | -1.2 (-4.9 to 2.5) | 0.53 |
